# Home Knowledge

**Published:** 1887-09

**Authors:** 


Home Knowledge. Edited by Robert A. Gunn. Published by Home Knowledge Association, of New York.

This is a monthly journal of valuable hints for the family upon all sorts of subjects, but especially health. It is a new publication, the copy now before us being the third number ever issued. Looking at its table of contents we notice, among other good articles, "Health Hints to Travelers in Mexico," "The New Education," "Bathing," "Kindergarten," "Circumstantial Evidence," "Ventilation." "Power of Music," "Aids to Beauty," etc. Its price is moderate—two dollars per year. The number before us contains sixty pages.

